# Supplementary material for: Five decades (1972–2020) of zooplankton monitoring in the upper San Francisco Estuary
Source: PLoS One. 2022 Mar 14;17(3):e0265402. doi: 10.1371/journal.pone.0265402 (PMC8920236; doi:10.1371/journal.pone.0265402)
Supplement: Attachment — Submitted filename: Bashevkin et al. 2021 PLoS One review.docx [file pone.0265402.s001.docx]

Review of Manuscript PONE-D-21-19296 by S. M. Bashevkin, R. Hartman, M. Thomas, A. Barros, C. E. Burdi, A. Hennessy, T. Tempel, & KI. Kayfetz entitled: “Five decades (1972-2020) of zooplankton monitoring in the upper San Francisco Estuary”, submitted to PLoS One.

I have reviewed several dozen manuscripts yearly over the last few decades, for scores of different journals, and by authors from close to a hundred countries. This is one of the strangest manuscripts that I have ever reviewed. It is not acceptable for publication in its present form, in PLoS One or any other reputable scientific journal. Specific criticisms are presented below.

While the abstract engages in bragging about how this is the “longest available dataset (by 15 years) of estuarine zooplankton abundance worldwide,” except for 3 figures, the manuscript presents NO DATA on zooplankton patterns! It reads like a technical report to a funding agency rather than a scientific paper. Further, the manuscript is not well-written, particularly for authors who are presumably writing in their first language. Also, the statement that “long-term zooplankton datasets from estuaries are less common” ignores several unmentioned estuarine zooplankton datasets in published papers that should have been cited (see recent issues of Journal of Plankton Research and papers cited therein). In short, this manuscript appears to have been written by authors who are inexperienced in writing scientific papers. While producing the long-term dataset is certainly commendable, its presentation deserves better than this manuscript, which is a disservice to the authors, and their colleagues and predecessors who devoted considerable effort to collecting and analyzing the data.

In terms of writing, there is inconsistency in the hyphenation of double-word adjectives, and the same words, or combinations of words, are frequently presented differently, and often incorrectly (“under sampled” versus “undersampled”, “lifecycle”, “timeseries”). In the tables, abbreviations for surveys should be given in the table legend of each table, not just once in the text. Pronouns that begin sentences (such as “They”) should refer to the last noun used in the previous sentence, not one used before the last noun. Slang such as “lab” instead of “laboratory” should be avoided. Descriptions of sampling methodology should be written in past tense (although the project appears to be ongoing, the data for this manuscript ended in 2020).

The length of the introductory material should be greatly reduced. The Introduction begins on p. 2, with “Background and Motivation” (p. 2-7), “Monitoring overview” (p. 7-10, “Dataset description and access” (p. 10-14), followed by “Methods” (with various subtitles) from p. 14-31. THERE IS NO “RESULTS” SECTION. The manuscript then concludes with sections for “Discussion” (p. 32-34), “References (p. 34-42), and several figures.

I am a producer of, and big fan of long-term investigations of plankton patterns. Thus, when I was asked to review this manuscript, I was looking forward to learning something new. I was disappointed in that, and somewhat irritated to have had to waste my time reading something so amateurish.
