# Supplementary material for: Five decades (1972–2020) of zooplankton monitoring in the upper San Francisco Estuary
Source: PLoS One. 2022 Mar 14;17(3):e0265402. doi: 10.1371/journal.pone.0265402 (PMC8920236; doi:10.1371/journal.pone.0265402)
Supplement: Attachment — Submitted filename: Responses to reviewers PLOS ONE.docx [file pone.0265402.s002.docx]

**Dear Dr. Miquel Vall-llosera Camps,**

**Since you have indicated over email that the only comments that we must respond to are those related to “Citation of datasets published in recent papers, as mentioned by Reviewer#2 and #3” I have edited the reviews from those reviewers to only include those points, and avoid pasting personal insults back into this response. Our responses to just those points are included below.**

**Reviewer #2**: Also, the statement that “long-term zooplankton datasets from estuaries are less common” ignores several unmentioned estuarine zooplankton datasets in published papers that should have been cited (see recent issues of Journal of Plankton Research and papers cited therein).

**We have extensively searched through the last 5 issues of the Journal of Plankton Research and only found 3 articles that could possibly be related to long-term estuarine zooplankton monitoring. Dudeck et al. 2021 (“Winter zooplankton dynamics in the English Channel and southern North Sea: trends and drivers from 1991 to 2013”) was excluded because the only data we could find for their reported data source included solely herring larval abundance and no other taxa (**[**https://eggsandlarvae.ices.dk/Download.aspx**](https://eggsandlarvae.ices.dk/Download.aspx)**, set survey in the filters to “IHLS”). Smith et al. 2012 (“*Acartia arbruta* (previously *A. tonsa*) in British Columbia: a bioindicator of climate-driven ecosystem variability in the northeast Pacific Ocean”) was excluded after researching the data source (Line P cruises by DFO, Canada) and discovering that samples were collected from the outer western coast of Vancouver Island, which is marine, not estuarine. Lastly, Monteiro et al. 2021 (“Long-term changes of ichthyoplankton communities in an Iberian estuary are driven by varying hydrodynamic conditions”) was excluded because we could find no evidence of the sampling program online.**

**However, to expand our search further we also searched for the term “estuary zooplankton” in the google data search (**[**https://datasetsearch.research.google.com/search?query=estuary%20zooplankton%20&docid=L2cvMTFqY2tmYjFwbg%3D%3D**](https://datasetsearch.research.google.com/search?query=estuary%20zooplankton%20&docid=L2cvMTFqY2tmYjFwbg%3D%3D)**) which returned >100 results. We looked through every result from that search for estuarine zooplankton datasets with >10 years of data (since the focus was on long-term monitoring, not short-term studies) and sampling of the zooplankton community instead of just a select few taxa (since the focus was on zooplankton monitoring, not monitoring of select species). From these results, we found 3 additional estuarine long-term monitoring datasets that we now mention in the paper (St. Lawrence Estuary, Canada; Bristol Channel, UK; Kongsfjorden, Norway).**

**These additional searches are in addition to the extensive searching we did for the first draft of the paper, which was focused on identifying other open-source published estuarine zooplankton monitoring datasets. For this search, we used targeted online searches in addition to inspecting every output of a search for the term “estuary zooplankton” in the DataONE network of data repositories.**

**Despite the additional zooplankton datasets we found, our statement that “long-term zooplankton datasets from estuaries are less common, and mostly of shorter time scales or smaller spatial scope” still holds true. No zooplankton scientist would disagree that marine and lake zooplankton have much more extensive monitoring programs. We could go into further detail on this, but believe that would distract from the purpose of the paper, which is to describe our specific dataset, not conduct a systematic review of all zooplankton monitoring datasets from lakes, oceans, and estuaries.**

In terms of writing, there is inconsistency in the hyphenation of double-word adjectives, and the same words, or combinations of words, are frequently presented differently, and often incorrectly (“under sampled” versus “undersampled”, “lifecycle”, “timeseries”). In the tables, abbreviations for surveys should be given in the table legend of each table, not just once in the text. Pronouns that begin sentences (such as “They”) should refer to the last noun used in the previous sentence, not one used before the last noun. Slang such as “lab” instead of “laboratory” should be avoided. Descriptions of sampling methodology should be written in past tense (although the project appears to be ongoing, the data for this manuscript ended in 2020).

**The word “undersampled” is only included in that form in the name of the data table “undersampled.csv”. It is common practice to remove spaces from file names. The words “life cycle” with a space between the two words never appear in our manuscript text. We have corrected all instances of “timeseries” to “time series.” We have added definitions of the survey acronyms to the appropriate tables. We have clarified ambiguous pronouns throughout the text. We have replaced all “lab” with “laboratory.” Sampling is ongoing as is identified in the text, so the methodology is described in present tense. If PLOS ONE has a style guide mandating past tense for all methodology, we are willing to change this, but would prefer present tense.**

**Reviewer #3**: good and important work to put the different data sets together. In the abstract, line 30, "what make the fishes important?" (commercially important?).

**We describe in the introduction why these fish are important, there is not space in the abstract to do so since we are at the word limit and would prefer to keep more words devoted to the zooplankton dataset than the fish species. Some species are commercially important and some are classified as endangered under Federal and State endangered species laws, with partial but not complete overlap between those two categories of importance.**

Then the UK people have similar data sets from around their islands and inland lakes. And Hawaii has the Hawaii Ocean Time-series (HOT) from their station ALOHA.

**Please see our response to reviewer 2 above for our search for additional estuarine zooplankton monitoring datasets and the new additions. We found one additional dataset from the UK. We are focused on estuarine datasets as comparisons, so we did not include the Hawaii ocean time series.**

One day one can find correlations like:

North–South shifts of the Gulf Stream and their climatic connection with the abundance of zooplankton in the UK and its surrounding seas --- Arnold H. Taylor
ICES Journal of Marine Science, Volume 52, Issue 3-4, June 1995, Pages 711–721, <https://doi.org/10.1016/1054-3139(95)80084-0>

**We agree that correlations between long-term zooplankton data and climate would be very interesting and recommend climate change as a topic of further research in the “Data Use and Recommendations for Reuse” section.**
