# Supplementary material for: Five decades (1972–2020) of zooplankton monitoring in the upper San Francisco Estuary
Source: PLoS One. 2022 Mar 14;17(3):e0265402. doi: 10.1371/journal.pone.0265402 (PMC8920236; doi:10.1371/journal.pone.0265402)
Supplement: Attachment — Submitted filename: reviwer comment - PONE-D-21-19296R2.docx [file pone.0265402.s004.docx]

**PONE-D-21-19296R2 - Five decades (1972-2020) of zooplankton monitoring in the upper San Francisco Estuary**

This manuscript presents the dataset of estuarine zooplankton abundance worldwide. This manuscript is very informative, the background is well written to set the premises of the dataset. The methodology is well explained. I also appreciate the inclusion of micro-zooplankton; these organisms are ecologically important but are often understudied. This dataset will be handy for ecological synthesis and ecosystem modellers. I recommend this manuscript for publication. However, I have some suggestions and queries

1. Figure 1. increase the x axis and y axis font size, the sampling points can be more well defined.
2. Include a line or two about the knowledge gap and future recommendations.
3. Rainfall plays huge role in the estuarine ecosystem, why was there no data about rainfall?
