# Supplementary material for: Five decades (1972–2020) of zooplankton monitoring in the upper San Francisco Estuary
Source: PLoS One. 2022 Mar 14;17(3):e0265402. doi: 10.1371/journal.pone.0265402 (PMC8920236; doi:10.1371/journal.pone.0265402)
Supplement: Attachment — Submitted filename: Response to reviewers V5.docx [file pone.0265402.s005.docx]

**PONE-D-21-19296R2 - Five decades (1972-2020) of zooplankton monitoring in the upper San Francisco Estuary**

**Reviewer #4**

This manuscript presents the dataset of estuarine zooplankton abundance worldwide. This manuscript is very informative, the background is well written to set the premises of the dataset. The methodology is well explained. I also appreciate the inclusion of micro-zooplankton; these organisms are ecologically important but are often understudied. This dataset will be handy for ecological synthesis and ecosystem modellers. I recommend this manuscript for publication. However, I have some suggestions and queries

**Thank you!**

1. Figure 1. increase the x axis and y axis font size, the sampling points can be more well defined.

**We have increased the axis font size and increased the size of the sampling points to make them better defined.**

2. Include a line or two about the knowledge gap and future recommendations.

**We have expanded the final paragraph of the paper (before the acknowledgements) where we talk about knowledge gaps and recommendations for use of this dataset. We have also made it clearer that this paragraph is related to knowledge gaps and linked the knowledge gaps more closely to management needs.**

3. Rainfall plays huge role in the estuarine ecosystem, why was there no data about rainfall?

**This dataset only includes data from the zooplankton surveys and these boat-based field surveys do not collect precipitation data. However, we have added information to the “Comparison with existing datasets” section with information on hydrology datasets (including rainfall) from the same region that could be used in tandem with our zooplankton dataset for users interested in these connections.**

**Reviewer #5**

Although, one of the reviewers talked about correlating the dataset with climate conditions, which can be done as another work. but I was at least expecting to see an interpretation of the result/data presented, or what the data show.

**A data paper is meant to describe a dataset, not interpret it. The interpretation would have to be a separate research paper. Since this dataset is so large, we could not investigate the effects of climate conditions on zooplankton in a simple manner that would fit within the confines of this paper. That analysis would be its own research paper (or a series of related studies) since it would involve complex statistical modeling. However, we did add more discussion of this future direction in the final paragraph of the paper.**
